# Cross-cultural adaptation and validation of the PedsQL™ stem cell transplant module in China: A methodological and cross-sectional study

**DOI:** 10.3389/fped.2022.964261

**Published:** 2023-01-05

**Authors:** Yuting Wei, Heng Zhang, Xiaowen Qian, Yao Xue, Liucheng Rong, Yaping Wang, Wenjin Jiang, Rufeng Lin, Shifeng Lu, Xiaowen Zhai, Yongjun Fang

**Affiliations:** ^1^ Department of Hematology and Oncology, Children’s Hospital of Nanjing Medical University, Nanjing, China; ^2^ Department of Hematology and Oncology, National Children’s Medical Center, Children’s Hospital of Fudan University, Shanghai, China

**Keywords:** quality of life (QoL), reliability, validity, hematopoietic stem cell transplantation (HCST), pediatric

## Abstract

**Background:**

Hematopoietic stem cell transplantation (HSCT), as a mature technology, has significantly improved the survival rate of children. However, there lack efficient scales to assess the quality of life (QoL) of children with HSCT in China, which has important implications in the care of this population. This study aimed to translate the original English Pediatric Quality of Life Inventory™ (PedsQL™) Stem Cell Transplant Module into a Chinese mandarin version, and evaluate its reliability.

**Methods:**

Children of ages 2–18 years who had received HSCT at Children's Hospital of Nanjing Medical University and Children's Hospital of Fudan University were recruited. Children or their parents were asked to fill the PedsQL™ 4.0 Generic Core Scales, PedsQL™ Stem Cell Transplant Module, and PedsQL™ Family Information Form. Feasibility was evaluated by completion rate and the percentage of missing items, reliability by the internal consistency and test-retest reliability, and validity by factor analysis and correlation analysis between the scores of total scale and each dimension.

**Results:**

A total of 120 children (mean age 6.37, SD = 3.674) and some parents were included. A low percentage of items were missed in returned reports. Cronbach's alpha coefficient reached 0.70 in the majority of dimensions of both child self-report and parent proxy-report. Test-retest reliability was 0.685 in parents' forms and 0.765 in child's forms. Eight factors were extracted, with a cumulative contribution rate of 74.54%. The correlation between PedsQL™ 4.0 and Transplant Module was 0.748 for children self-report and 0.808 for parent proxy-report.

**Conclusions:**

This study provides evidence that the Chinese mandarin version of the PedsQL™ Stem Cell Transplant is feasible, reliable and valid in evaluating the QoL of Chinese children after HSCT.

## 1. Introduction

Hematopoietic stem cell transplantation (HSCT) has exhibited its high effectiveness in managing the symptoms and improving the survival of malignant and malignant disorders (1, 2). However, patients' quality of life (QoL), either physical or psychosocial, has been less considered during this treatment. QoL may be impaired by long-term adverse events after HSCT, including chronic graft-vs.-host disease (cGVHD), infection, pain, secondary cancer, organ dysfunction, fertility decline and systemic diseases (3, 4). It has been reported that 90.2% of children develop at least one adverse event after HSCT (5), with cGVHD as the most dangerous (6, 7).

Health-related Quality of Life (HRQoL) is assessed by objective parameters (e.g., those about organ function) and subjective parameters (e.g., those self-reported) (8, 9). Evaluation of HRQoL is of high clinical and scientific significance. Therefore, the tool to evaluate HRQoL must be practical and multidimensional. Several tools have been invented for adult patients receiving HSCT, such as the 36-Item Short-Form Health Survey (SF-36) and Function Assessment of Cancer Therapy-Bone Marrow Transplantation (FACT-BMT) (10, 11). However, the instrument for HSCT-treated children lack in China (12).

The Pediatric Quality of Life Inventory™ (PedsQL™) is authoritative to measure the HRQoL of children aged 2–18 years (13, 14). This instrument consists of a generic core scale, a disease-specific module, and other condition-specific modules, most of which have been established with satisfactory reliability and validity in China (15–18). PedsQL™ 3.0 Transplant Module is used to evaluate the HRQoL of patients after HSCT in China, but it focuses on those receiving solid organ transplantation (19), a surgery highly different from HSCT. However, the generic PedsQL and the PedsQL™ 3.0 Transplant Module are not cGVHD-specific, and cannot yield detailed information about factors that impact on the QoL of pediatric patients with cGVHD. To be specific to HSCT and cGVHD, the PedsQL™ Stem Cell Transplant Module was developed in 2014 by James W. Varni et al. (20). The current study was to cross-culturally adapt the PedsQL™ Stem Cell Transplant Module, and evaluate the feasibility, reliability and validity of this version in a pediatric population with HSCT.

## 2. Methods

### 2.1. Participants

The cross-sectional study was carried out from June 2021 to December 2021 in Nanjing and Shanghai, China. Children who had received HSCT at Children's Hospital of Nanjing Medical University and Children's Hospital of Fudan University were recruited.

The inclusion criteria were: ≥2 years old, native Chinese, written informed consent from parents or patients ≥8 years old. Meanwhile, excluded were children who had other chronic diseases or significant developmental disorders severe enough to affect their HRQoL, children who or whose parents had cognitive difficulty or illiteracy, or were reluctant to participate in the study. Finally, 120 children and some parents were included, meeting the minimum size of this reliability analysis.

### 2.2. Analytical tools

#### 2.2.1. PedsQL™ 4.0 generic core scales

HRQoL of the HSCT-treated children was assessed according to the PedsQL™ Generic Core Scales. The PedsQL™ 4.0 Generic Core Scales were developed by James W. Varni et al. in 1999 to measure the HRQoL of children and adolescents aged 2–18 years. These scales have demonstrated good validity and reliability in Chinese populations. In the present study, the scales were translated in Chinese mandarin. The PedsQL™ 4.0 includes child self-report and parent proxy-report formats. Child self-report is administered to children ages 5–7, 8–12, and 13–18 years, while parent proxy-report to those ages 2–4, 5–7, 8–12, and 13–18. The 23-item multidimensional PedsQL™ 4.0 compasses 4 scales: (1) Physical Functioning (8 items), (2) Emotional Functioning (5 items), (3) Social Functioning (5 items), and (4) School Functioning (5 items). The items are set to question on the frequency of problems that occurred during the past months, and each question is answered by one of the five Likert response options, including 0 (never), 1 (rarely), 2 (sometimes), 3 (often) and 4 (almost always). The scores at both item and scale levels are converted according to a 0–100 point scale, with higher scores indicating better functioning.

#### 2.2.2. PedsQL™ stem cell transplant module

HRQoL of the HSCT-treated children was assessed specifically according to the PedsQL™ Stem Cell Transplant Module. The PedsQL™ Stem Cell Transplant Module encompasses eight subscales: (1) Pain and hurt (2 items), (2) Fatigue (5 items), (3) Nausea (5 items), (4) Worry/anxiety about disease/treatment (12 items), (5) Nutritional problems (5 items), (6) Thinking/remembering (4 items), (7) Communication about disease/treatment (3 items), (8) Other complains-specifically cGVHD-related problems (6 items). Child self-report is administered to children ages 8–12 and 13–18 years, and parent proxy-report to children ages 2–4, 5–7, 8–12, and 13–18 years. The items and scoring method of the Transplant Module are similar to those of the PedsQL™ 4.0 Generic Core Scales. Higher scores indicate better HRQoL.

#### 2.2.3. PedsQL™ family information form

The PedsQL™ Family Information Form is completed by parents to obtain demographic information about one child and his/her parents, pertaining to the child's gender, birth date, disease, and the parent's occupation and education, family income, and payment methods for their child's healthcare, etc.

### 2.3. Translation and pre-test

**Figure 1:**
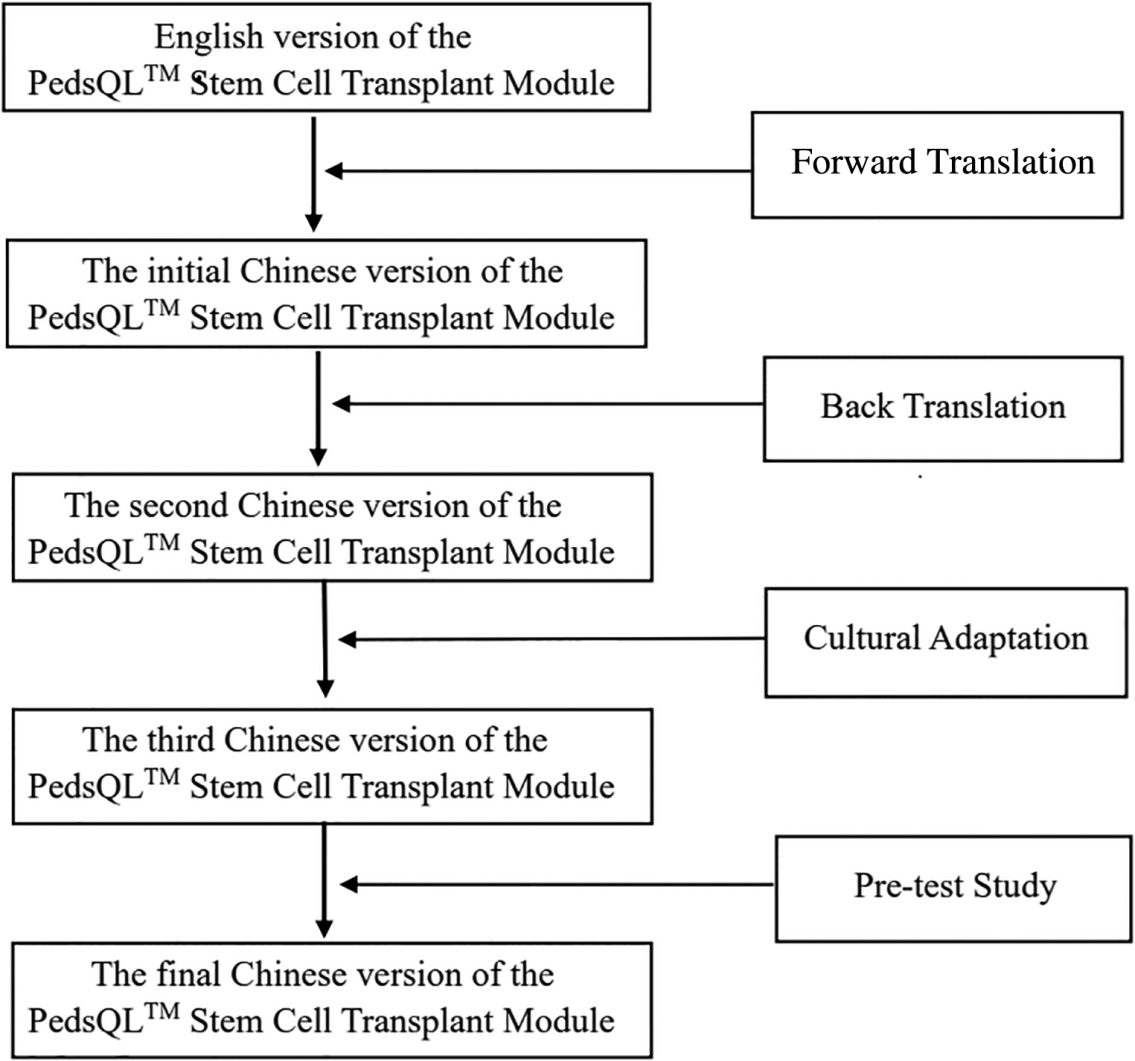
The translation procedure of the PedsQL™ stem cell transplant module.

Under the permission from Professor James W. Varni, the copyright holder of the PedsQL™, we translated it into a Chinese version in accordance with the MAPI Research Trust linguistic validation guidelines, including forward translation, back translation, and patient testing (21, 22). The processes are as follows:

• The first step was forward translation. During this stage, the English PedsQL™ was independently translated into a Chinese version by two local professional translators, who are native Chinese Mandarin speakers and fluent in English language. A single reconciled Chinese version was created using the two translated versions through consensus.
• The second step was back translation, from the first Chinese version to the English version, performed by two native Chinese pediatricians who are rich experience in QoL research, fluent in English, and have worked in English-speaking countries for at least three years. Without access to the initial version of the PedsQL™ Stem Cell Transplant Module, both pediatricians revised the version, then the back-translated version was compared to the original version by an expert team, composed of pediatricians, health-quality life researchers and translators. Errors were commented and resolved to form the Second Chinese version. After cultural adaptation, the experts reached a consensus, laying out the third Chinese version.
• Then, 20 children, who received HSCT in the custody of their parents, were recruited to complete the pre-test through face-to-face interviews. They were asked about whether the terms used in the Chinese version were understandable and acceptable. Based on their answers and feedback, we identified problems and modified the presentation. The final Chinese version of the PedsQL™ Stem Cell Transplant Module was developed and used in the following steps of the study. The translation procedures are shown in Figure 1.

### 2.4. Data collection

Participants were informed of the purpose and procedure of the study. Age-related questions were printed and handed out to parents and children to measure HRQoL (PedsQL™ Stem Cell Transplant Module, PedsQL™ 4.0 Generic Core Scales, PedsQL™ Family Information Form). The patients discharged filled the questionnaires by phone. They were asked to fill the questionnaires independently during hospitalization or outpatient care. Any trouble of the participant in understanding the question was resolved by the investigators. Test-retest reliability was assessed at Children's Hospital of Nanjing Medical University. Two to three weeks following the first assessment, children and their parents again completed the PedsQL™ stem cell transplant module. Finally, double data entry was performed to ensure accuracy. The data were invalid and excluded if 25% or more of the items were not answered.

### 2.5. Statistical analysis

All data were analyzed by Statistical Packages of Social Sciences (SPSS) software packages (version 23; IBM Corp, Armonk, NY, United States). Demographic and clinical characteristics were presented as mean and standard deviation (SD) or percentage (%). The feasibility of the scale was evaluated by completion rate and the percentage of missing items. The reliability of the questionnaire was mainly assessed by internal consistency and test-retest reliability. Cronbach's coefficient alpha was used to evaluate the internal consistency, and a value above 0.7 was acceptable (23). Test-retest reliability was used to measure the consistency of the scale's performance at different time points. In this study, we used the intra-class correlation coefficient (ICC) between the initial test and retest results to determine the reliability of the scale, with an ICC above 0.5 indicating moderate (23–25). To analyze the construct and content validity, principal factor analysis and correlation analysis between the scores of the total scale and each dimension. Additionally, the assessment of criterion validity was based on Pearson's correlation between the PedsQL™ 4.0 and Stem Cell Transplant Module (26).

## 3. Results

### 3.1. Characteristics

A total of 120 subjects (mean age 6.37 years, SD = 3.674; 71 males [59.2%]; 49 females [40.8%]) were elligable for the study. Parent proxy-report forms of PedsQL™ Stem Cell Transplant Module were completed by 120 parents of children aged from 2 to 18 years, and child self-report forms by 42 children aged from 8 to 18 years (except for 78 children aged between 2 and 7 years). Among the parents enrolled in the study, 87 mothers (72.5%) answered the questionnaires alone and 31 fathers (25.8%). Further details are summarized in Table 1. The clinical characteristics of included children are exhibited in Table 2. Forty patients (33.3%) were diagnosed with malignant diseases and 80 (66.7%) with nonmalignant diseases. The diagnoses included acute leukemia, lymphoma, hemophagocytic syndrome, neuroblastoma, aplastic anemia, immunodeficiency disease, chronic granulomatous. The most common donor was matched unrelated donor (60.8%). Of the 120 patients, 84 (70.0%) received the MAC regimen, and 36 (30.0%) RIC as conditioning regimens.

**Table 1:** Demographic characteristics of the sample.

Demographic Characteristics	Children (*N* = 120)	
	*N*	%
Mean age (years)	6.37 (SD = 3.674)	
Age (years)		
2–4	48	40.0
5–7	30	25.0
8–12	33	27.5
13–18	9	7.5
Gender		
Male	71	59.2
Female	49	40.8
Respondent		
Mother	87	72.5
Father	31	25.8
Others	2	1.7
Education level of mother		
Primary school	8	6.7
Secondary school	39	32.5
High school	26	21.7
University or above	45	37.5
Others	2	1.6
Occupation of mother		
Employed	68	56.6
Unemployed	50	41.7
Others	2	1.7
Education level of father		
Primary school	4	3.3
Secondary school	42	35.0
High school	32	26.7
University or above	37	34.1
Others	1	0.8
Occupation of father		
Employed	105	87.5
Unemployed	14	11.7
Others	1	0.8
Family intimacy		
Well	74	61.7
Good	37	30.8
Average	8	6.7
Others	1	0.8
Family income		
Intermediate upper	10	8.3
Intermediate mid	63	52.5
Intermediate lower	46	38.4
Others	1	0.8

**Table 2:** Clinical characteristics of the sample.

Clinical Characteristics		Children (*N* = 120)	
		*N*	%
Diagnosis	**Malignant diseases**	40	33.3
	Acute myeloid leukemia	13	10.8
	Acute lymphoid leukemia	3	2.5
	Acute biphenotypic leukemia	2	1.7
	Chronic myeloid leukemia	3	2.5
	Juvenile granular monocytic	2	1.7
	Lymphoma	5	4.1
	Hemophagocytic syndrome	5	4.1
	Neuroblastoma	5	4.1
	Rhabdomyosarcoma	2	1.7
	**Nonmalignant diseases**	80	66.7
	Aplastic anemia	19	15.8
	Immunodeficiency disease	18	15.0
	Chronic granulomatous	16	13.3
	Inflammatoty bowel disease	9	7.5
	Thalassemia	3	2.5
	Mucopolysaccharidosis	2	1.7
	Adrenoleukodystrophy	2	1.7
	Other congenital diseases	11	9.2
Type of transplant	Matched Related	47	39.2
	Matched Unrelated	73	60.8
Conditioning regimens	Myeloablative Conditioning regimens (MAC)	84	70.0
	Reduced intensity Conditioning regimens (RIC)	36	30.0

### 3.2. Cross-cultural adaptation and pre-test

Several items in the English version were modified to adapt to Chinese patients. To reduce negative effects on the communication with children's parents, we try to avoid sensitive words when talking about children's condition. For example, words like “tumor” or “cancer” were replaced by “illness” in the Chinese version. “Difficulties in sleeping through” was changed to “Difficulties in getting a good night's sleep”; “Worry about returning to normal life” was adapted to “Worry about failing to return to normal life”. During the pre-test, the children and parents agreed that the Chinese version was easy to understand, and they had no difficulties in filling the questionnaire.

The PedsQL™ Stem Cell Transplant Module provides items about the developmental and understanding for children of a wide range of ages. Given the lower cognitive ability of the younger children, some questions were omitted from the younger questionnaire. Therefore, there are differences in some items. For example, self-report of children younger than 8 years was not included in the Chinese version. In addition, the item “Worry/anxiety about disease/treatment” showed the most significant between-version difference. Children older than 8 years were asked to repond to “worry about side effects from medical treatment”, “worry about disease recurrence”, “worry about not returning to normal life”, “think about having kids”, and so on. There was no difference in other items.

### 3.3. Means and standard deviations

The means and standard deviations of scores are listed in Table 3. For the PedsQL™ 4.0 scale, the mean child self-report score (72.88, SD = 20.54) was significantly higher than the mean parent proxy-report score (68.85, SD = 22.64) in the entire group. For the Stem Cell Transplant Module, the mean child-report score (74.66, SD = 11.09) was slightly higher than the mean parent proxy-report score (70.38, SD = 18.30).

**Table 3:** The PedsQL™ 4.0 generic core scales and stem cell transplant module scores.

Scale		Child self-report		Parent proxy-report	
		Mean	SD	Mean	SD
PedsQL™ 4.0	**Total score**	72.88	20.54	68.85	22.64
	Physical functioning	69.01	25.26	65.41	27.92
	Emotion functioning	78.61	20.98	74.50	22.81
	Social functioning	77.77	21.27	72.87	25.60
	School-related problems	68.00	26.25	62.42	31.13
PedsQL™ Stem Cell Transplant	**Total score**	74.66	11.09	70.38	18.30
	Pain and hurt	77.67	20.57	72.50	24.23
	Fatigue	76.66	16.32	70.83	23.73
	Nausea	77.08	15.95	75.26	21.36
	Worry about disease	70.65	14.79	60.30	27.10
	Nutritional problems	74.16	14.31	69.29	16.83
	Thinking	77.38	14.27	71.25	23.30
	Communication	70.63	21.95	69.23	29.20
	Others	77.97	11.90	74.30	19.47

SD, standard deviation.

### 3.4. Feasibility

In this study, 120 parent proxy-report forms and 42 children self-report forms were sent out, all returned. Because a considerable number of out-spatients were out of school age, the missed items were mainly about “School functioning”. Additionally, none patients aged ≥8 years or parents had difficulties in understanding the items. The majority of the subjects did not require assistance in completing the questionnaire, and it took them an average of 10 min to complete.

### 3.5. Reliability

The internal consistency reliability measured by Cronbach's alpha coefficient for the PedsQL™ Stem Cell Transplant Module is presented in Table 4. Cronbach's alpha coefficient of total score approached 0.70 in both children self-report and parent proxy-report. Cronbach's alpha coefficient ranged from 0.608 to 0.956 in child self-report and 0.93–0.98 in parent's report.

**Table 4:** Internal consistency reliability of the stem cell transplant module.

Dimensions	Age group (years)			
	Toddler (2–4)	Young child (5–7)	Child (8–12)	Adolescent (13–18)
**Parent proxy-report**	***N* = 48**	***N* = 30**	***N* = 33**	***N* = 9**
Total score	0.959	0.980	0.941	0.976
Pain and hurt	0.637	0.630	0.768	0.808
Fatigue	0.934	0.955	0.895	0.903
Nausea	0.832	0.934	0.872	0.909
Worry about disease	0.843	0.9767	0.882	0.966
Nutritional problems	0.731	0.826	0.621	0.712
Thinking	0.963	0.903	0.639	0.830
Communication	0.989	0.882	0.862	0.867
Others	0.842	0.887	0.814	0.732
**Child self-report**	** **	** **	***N* = 33**	***N* = 9**
Total score			0.913	0.956
Pain and hurt			0.620	0.948
Fatigue			0.817	0.770
Nausea			0.778	0.951
Worry about disease			0.891	0.930
Nutritional problems			0.668	0.755
Thinking			0.915	0.695
Communication			0.871	0.608
Others			0.806	0.793

Values denote Cronbach's Alpha Coefficient.

Test-retest reliability was estimated in 29 patients who completed the initial test. The average interval between the initial test and the retest was 15.2 days (SD = 2.0 days). As shown in Table 5, the ICC value of the whole scale was 0.685 (*P *< 0.001) in parent-proxy report and 0.765 (*P *< 0.001) in child self-report.

**Table 5:** Test-retest reliability of the stem cell transplant module.

Dimension	Child self-report (*N* = 16)		Parent proxy-report (*N* = 29)	
	ICC	*P*	ICC	*P*
Total score	0.765	<0.001	0.685	<0.001
Pain and hurt	0.611	<0.001	0.811	<0.001
Fatigue	0.614	<0.001	0.742	<0.001
Nausea	0.756	<0.001	0.568	<0.001
Worry about disease	0.595	<0.001	0.667	<0.001
Nutritional problems	0.662	<0.001	0.636	<0.001
Thinking	0.787	<0.001	0.699	<0.001
Communication	0.835	<0.001	0.705	<0.001
Others	0.723	<0.001	0.556	<0.001

ICC, intra-calss correlation coefficient.

### 3.6. Validity

**Figure 2:**
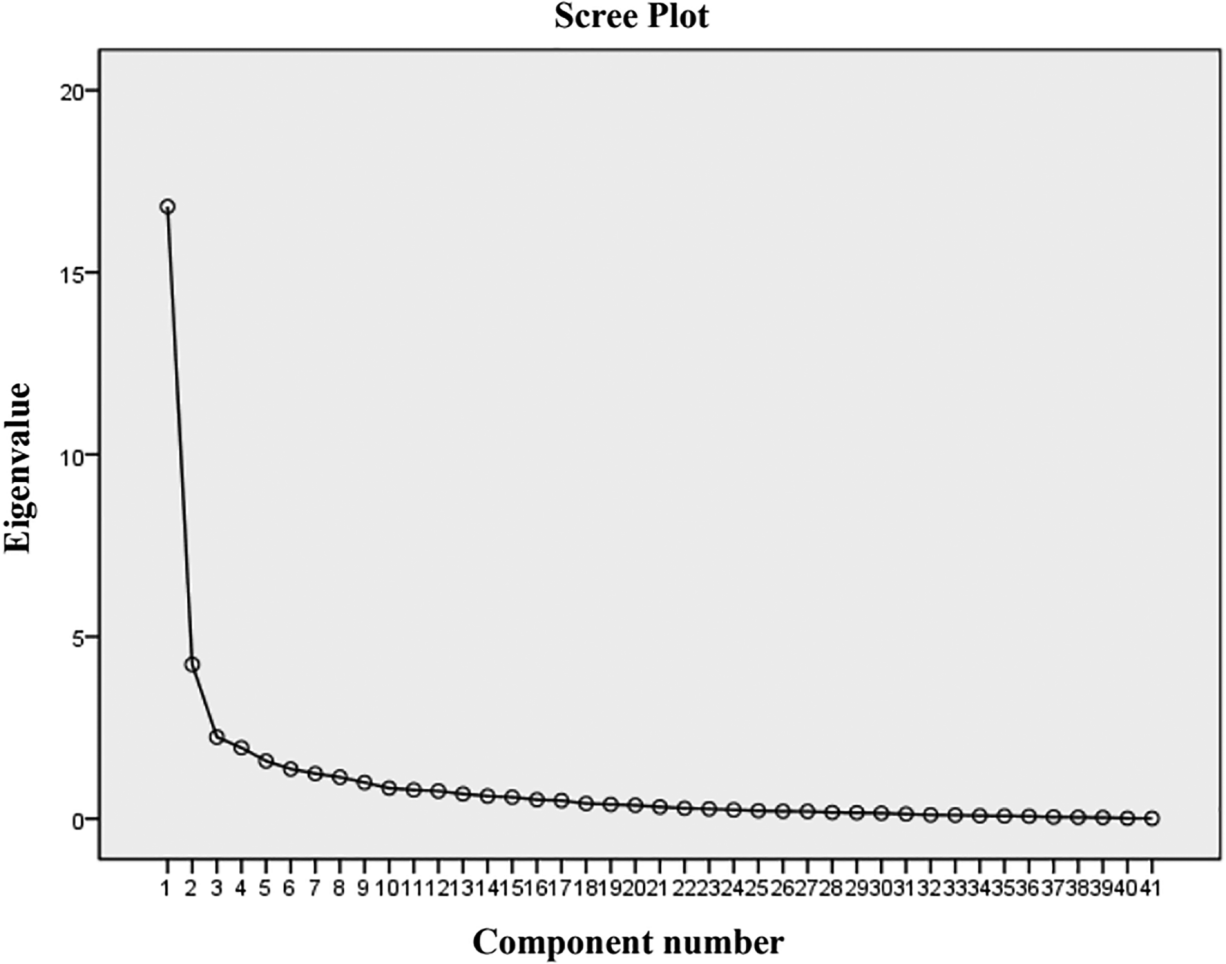
Scree plot of the 41-item PedsQL™ stem cell transplant scale. There were eight factors with an eigenvalue greater than 1.

Exploratory factor analysis was utilized to evalute the construct validity of the scale. The results of the principal component analysis for the parent proxy-report of the Cell Transplant Module are presented in Table 6. An eigenvalue cutoff of 1.0 was extracted as the common factor, resulting in an eight-factor solution for parent proxy-report, and their cumulative variance contribution rate was 74.54%, which indicated an excellent extraction effect. The scree plot also identified eight factors that could be extracted (Figure 2). Content validity was evaluated by Spearman's rank correlation analysis of the whole scale and each dimension. The child self-report is presented in Table 7 and parent proxy-report in Table 8. The correlation coefficients ranged from 0.222 to 0.849 for child self-report and from 0.428 to 0.902 for parent proxy-report. Most interscale correlations were significant at 0.05.

**Table 6:** Factors extracted by principal component analysis.

Component	Initial eigenvalues			Extraction sums of squared loadings			Rotation sums of squared loadings		
	Total	% of variance	Cumulative %	Total	% of variance	Cumulative %	Total	% of variance	Cumulative %
**1**	16.807	40.992	40.992	16.807	40.992	40.992	7.209	17.583	17.583
**2**	4.235	10.330	51.322	4.235	10.330	51.322	6.313	15.398	32.981
**3**	2.243	5.470	56.792	2.243	5.470	56.792	3.983	9.714	42.695
**4**	1.949	4.753	61.545	1.949	4.753	61.545	3.178	7.751	50.446
**5**	1.584	3.864	65.409	1.584	3.864	65.409	2.767	6.749	57.195
**6**	1.366	3.331	68.740	1.366	3.331	68.740	2.637	6.433	63.627
**7**	1.239	3.022	71.762	1.239	3.022	71.762	2.302	5.616	69.243
**8**	1.139	2.778	74.540	1.139	2.778	74.540	2.172	5.297	74.540

Extraction method: principal component analysis.

**Table 7:** Correlation between total score of the PedsQL™ stem cell transplant module and each dimension *for child self-report.*

Dimension	1	2	3	4	5	6	7	8	9
1 Total score	1	0.678**	0.849**	0.664**	0.787**	0.651**	0.694**	0.494**	0.709**
2 Pain and hurt	0.678**	1	0.666**	0.412**	0.437**	0.458**	0.372*	0.237	0.467**
3 Fatigue	0.849**	0.666**	1	0.648**	0.582**	0.568**	0.467**	0.252	0.631**
4 Nausea	0.664**	0.412**	0.648**	1	0.330*	0.460**	0.427**	0.295	0.525**
5 Worry about disease	0.787**	0.437**	0.582**	0.330*	1	0.321*	0.479**	0.423**	0.545**
6 Nutritional problems	0.651**	0.458**	0.568**	0.460**	0.321*	1	0.455**	0.459**	0.378*
7 Thinking	0.694**	0.372*	0.467**	0.427**	0.479**	0.455**	1	0.592**	0.548**
8 Communication	0.494**	0.237	0.252	0.295	0.423**	0.459**	0.592**	1	0.222
9 Others	0.709**	0.467**	0.631**	0.525**	0.545**	0.378*	0.548**	0.222	1

Values denote Spearman's rank correlation coefficients.

* Correlation is significant at the 0.05 level.

** Correlation is significant at the 0.001 level.

**Table 8:** Correlation between total score of the PedsQL™ stem cell transplant module and each dimension for *parent proxy-report.*

Dimension	1	2	3	4	5	6	7	8	9
1 Total score	1	0.719**	0.862**	0.820**	0.693**	0.809**	0.770**	0.759**	0.902**
2 Pain and hurt	0.719**	1	0.687**	0.595**	0.507**	0.543**	0.498**	0.430**	0.637**
3 Fatigue	0.862**	0.687**	1	0.737**	0.511**	0.688**	0.559**	0.611**	0.766**
4 Nausea	0.820**	0.595**	0.737**	1	0.469**	0.628**	0.625**	0.543**	0.707**
5 Worry about disease	0.693**	0.507**	0.511**	0.469**	1	0.538**	0.480**	0.428**	0.540**
6 Nutritional problems	0.809**	0.543**	0.688**	0.628**	0.538**	1	0.639**	0.557**	0.733**
7 Thinking	0.770**	0.498**	0.559**	0.625**	0.480**	0.639**	1	0.568**	0.649**
8 Communication	0.759**	0.430**	0.611**	0.543**	0.428**	0.557**	0.568**	1	0.704**
9 Others	0.902**	0.637**	0.766**	0.707**	0.540**	0.733**	0.649**	0.704**	1

Values denote Spearman's rank correlation coefficients.

* Correlation is significant at the 0.05 level.

** Correlation is significant at the 0.001 level.

The Pearson's correlation between the PedsQL™ 4.0 and Stem Cell Transplant Module results was analyzed to examine the criterion validity of the scale. The results showed the correlation coefficient was 0.748 for child self-report and 0.808 for parent proxy-report. All correlations were statistically significant (*P *< 0.001). Additionally, a positive correlation was found between child self-report and parent proxy-report (*r* = 0.777, *P *< 0.001).

## 4. Discussion

In the present study, we formulated a Chinese mandarin version of the PedsQL™ Stem Cell Transplant Module through forward translation, back translation, and cross-cultural adaptation. The psychometric properties of the scale were developed in this study. To the best of our knowledge, this is the first cross-sectional study to demonstrate the feasibility, reliability and validity of the Chinese mandarin version of the PedsQL™ Stem Cell Transplant Module in a cohort of Chinese children after HSCT. The PedsQL™ Stem Cell Transplant Module has shown its strength in measuring the post-HSCT QoL of children and adolescents aged 2–18 years, especially those with cGVHD (20). Moreover, its child self-report and parent poxy-report can capture the information about the disease and treatment-related QoL of pediatric patients after HSCT.

In the present study, it did not take much time to complete the questionnaire in the Chinese version, which made it applicable in clinical practice. Few items on the Transplant Module were missed, suggesting that data regarding children's QoL was valid and the Chinese version was reliable. Additionally, we found that most of the missed items were about “School functioning”, indicating that children after HSCT recover slowly due to their weaker immunity and higher susceptibility to infection. This finding is comparable to that reported in the previous study (18, 20, 27). Not only the patients after HSCT, but the children with cancer after chemotherapy may miss school life due to clinic visits, hospitalizations, or intercurrent illnesses, which may explain why the children with HSCT reported a lower HRQoL than healthy children, especially in “School functioning”. The phenomenon also suggests that the children's eagnerness to back to school should be paid more attention by clinicians and families, and the hospital or communtiy should provide more support in this aspect.

Cronbach's alpha coefficient was used to examine internal consistency reliability. The alpha value above 0.7 indicated a high internal consistency. In the total scores of both children self-report and parent proxy-report of the PedsQL™ Stem Cell Transplant Module, Cronbach's alpha coefficient reached or exceeded 0.70, suggesting the total scale score has a good reliability and is suitable for analyzing the HRQL in clinical trials. The score of most dimensions also exceeded 0.7, but the score of “Pain and hurt” in parent proxy-report was below 0.7, which may be related to the small number of items in this dimension, and it also demonstrated that Cronbach's alpha coefficient was sensitive to the number of items and decrease with reduced items (28). In addition, the Cronbach's alpha coefficient for the child self-report in the group aged 13–18 years was low, which may be also due to the low number of patients included (*N* = 9). Previous studies showed that the score of “Nausea” and “Communication” in the child self-report was less than 0.7, which is inconsistent with our finding.

To avoid interference from memory loss and disease condition, it is recommended that the test-retest reliability evaluation should be performed at an appropriate time after the first investigation. In the present study, an interval of 2–3 weeks was set, and the re-test reliability was assessed by 29 patients. All scales for both parent proxy-report and child self-report showed good test-retest reliability, indicating that the results of Chinese mandarin version of the PedsQL™ Stem Cell Transplant Module are stable during a moderate term.

As can be expected, the results of the exploratory factor analysis supported the construct validity of the scales in the current study. Eight common factors were extracted, and showed a cumulative contribution rate of >70% in parent proxy-report, indicating the excellent extraction effect. This finding is consistent with that achieved by the original English version (20, 29). However, we did not complete the factor analysis in child self-report, because of the small number of children aged 8–18 years. Additionally, our findings also support that there was an excellent correlation between the PedsQL scores in parent proxy-report and child self-report, with the former lower the later. This has also been proven in previous research (15, 16, 27). The result may reflect that the parents' perceptions differ from the children's. One child's health may be differentially evaluated by self and his/her parents, who have different access to information and ability to interpret this information. The parents are more concerned about the outcomes of disease, but the children can adapt to their condition faster than their parents expcet. On the other hand, the understanding of parents on HRQoL is also an influencing factor. Therefore, it also validated the importance of pediatric patient inclusion in syoptom and HRQoL assessment. The correlation coefficients between child self-report dimensions stayed lower than those between parent proxy-report dimensions. One reason may be the small size of the chidren who completed the child self-report independently, so we will increase the sample size for further validation. Another reason is that children may hide their real attitudes regarding the communication domain, leading to a lower correlation coefficients between them. There are three items in “Communication” of the scale, including specific problems about “Communication with doctors, nurses and other people”. In our study, the child self-report was performed through face-to-face interviews; however, the children were prone to saying “no problems” in the inverview when confronted with a doctor. Compared to those of chidren, parents' perceptions would be more objective and realistic, suggesting the necessity of including chidren's parents in HRQoL assessment.

This cross-sectional study has several strengths, including a rigorous approach of constructing and assessing attributes, a wide range of age groups, and different types of transplantation and conditioning regimens of subjects. In addition, our study highlighted that the Chinese mandarin version of the PedsQL™ Stem Cell Transplant Module is reliable to evaluate the long-term effect of HSCT on Chinese-speaking children's QoL.

There are some limitations in this study. First, the sample size was not sufficient to make comparison between age groups. Children older than 8 years can finish the child self-report independently, while the average age of the subjects was 6.37 years in this study, propably bringing with age-related bias. Hence, the scale should be ascertained in further longitudinal clinical trials. Second, the participants were recruited from only two hospitals, which might bring about selection bias. Our findings should be validated in multiple centers throughout China.

On the other hand, the subjects in this study were included at different time points after HSCT, with the longest at up to 4 years. We have tentatively found a strong association between the post-HSCT time point and QoL, which requires further research. Therefore, we will carry out a cohort study of children with HSCT to dynamically assess changes in QoL and provide relevant clues for the care of children with HSCT.

## 5. Conclusion

The Chinese mandarin version of the PedsQL™ Stem Cell Transplant Module is feasible, reliable and valid to assess the QoL of children after HSCT. Modifications should be made before its wide clinical use.

## Data Availability

The original contributions presented in the study are included in the article/Supplementary Material, further inquiries can be directed to the corresponding author/s.
